# Ageism and Artificial Intelligence: Protocol for a Scoping Review

**DOI:** 10.2196/33211

**Published:** 2022-06-09

**Authors:** Charlene H Chu, Kathleen Leslie, Jiamin Shi, Rune Nyrup, Andria Bianchi, Shehroz S Khan, Samira Abbasgholizadeh Rahimi, Alexandra Lyn, Amanda Grenier

**Affiliations:** 1 Lawrence S Bloomberg Faculty of Nursing University of Toronto Toronto, ON Canada; 2 Institute for Life Course and Aging University of Toronto Toronto, ON Canada; 3 KITE Research Institute - Toronto Rehabilitation Institute University Health Network Toronto, ON Canada; 4 Faculty of Health Disciplines Athabasca University Athabasca, AB Canada; 5 Canadian Health Workforce Network Ottawa, ON Canada; 6 Dalla Lana School of Public Health University of Toronto Toronto, ON Canada; 7 Leverhulme Centre for the Future of Intelligence University of Cambridge Cambridge United Kingdom; 8 Department of Clinical and Organizational Ethics University Health Network Toronto, ON Canada; 9 Institute of Biomedical Engineering University of Toronto Toronto, ON Canada; 10 Department of Family Medicine McGill University Montreal, QC Canada; 11 Mila - Quebec AI Institute Montreal, QC Canada; 12 Lady Davis Institute for Medical Research Herzl Family Practice Centre Jewish General Hospital Montreal, QC Canada; 13 Factor-Inwentash Faculty of Social Work University of Toronto Toronto, ON Canada; 14 Baycrest Hospital Toronto, ON Canada

**Keywords:** artificial intelligence, ageism, age-related biases, gerontology, algorithms, search strategy, health database, human rights, ethics

## Abstract

**Background:**

Artificial intelligence (AI) has emerged as a major driver of technological development in the 21st century, yet little attention has been paid to algorithmic biases toward older adults.

**Objective:**

This paper documents the search strategy and process for a scoping review exploring how age-related bias is encoded or amplified in AI systems as well as the corresponding legal and ethical implications.

**Methods:**

The scoping review follows a 6-stage methodology framework developed by Arksey and O’Malley. The search strategy has been established in 6 databases. We will investigate the legal implications of ageism in AI by searching grey literature databases, targeted websites, and popular search engines and using an iterative search strategy. Studies meet the inclusion criteria if they are in English, peer-reviewed, available electronically in full text, and meet one of the following two additional criteria: (1) include “bias” related to AI in any application (eg, facial recognition) and (2) discuss bias related to the concept of old age or ageism. At least two reviewers will independently conduct the title, abstract, and full-text screening. Search results will be reported using the PRISMA-ScR (Preferred Reporting Items for Systematic Reviews and Meta-Analyses Extension for Scoping Reviews) reporting guideline. We will chart data on a structured form and conduct a thematic analysis to highlight the societal, legal, and ethical implications reported in the literature.

**Results:**

The database searches resulted in 7595 records when the searches were piloted in November 2021. The scoping review will be completed by December 2022.

**Conclusions:**

The findings will provide interdisciplinary insights into the extent of age-related bias in AI systems. The results will contribute foundational knowledge that can encourage multisectoral cooperation to ensure that AI is developed and deployed in a manner consistent with ethical values and human rights legislation as it relates to an older and aging population. We will publish the review findings in peer-reviewed journals and disseminate the key results with stakeholders via workshops and webinars.

**Trial Registration:**

OSF Registries AMG5P; https://osf.io/amg5p

**International Registered Report Identifier (IRRID):**

DERR1-10.2196/33211

## Introduction

Artificial intelligence (AI)—defined as “[the] designing and building of intelligent agents that receive percepts from the environment and take actions that affect that environment” [1]—has emerged as a major driver of technological development in the 21st century [2]. Although AI is often viewed as a neutral force, many widely deployed AI applications encompass the racial and gender biases that pervade society [3]. This is partly because AI models use input data that is mostly human-curated and thus are susceptible to encompass implicit and explicit bias as the basis for prediction—in other words, “bias in, bias out.” The following are some examples of AI bias. A widely used algorithm for population health management in the United States underestimated the health risks of Black patients due to their limited access to health care as a consequence of systemic racism [4]. Word2vec, a publicly available embedding algorithm, amplified the gender biases inherited from its training data by forming associations between words related to gender and occupation—in particular, “men” to “computer programmer” and “women” to “homemaker” [5]. Research also suggests that AI-driven algorithms show women fewer advertisements for high-paying jobs since these jobs have a historical context of being occupied by men [5].

An aging global population [6] brings new social challenges, most notably with regards to ageism and social exclusion. Ageism is an age-related bias that is conceptualized to include (1) prejudicial attitudes toward older adults and the process of aging, (2) discriminatory practices against older adults, or (3) institutionalized policies and social practices that foster the attitudes and actions in relation to (1) and (2) [7]. The World Health Organization recently published a policy brief entitled “Ageism in Artificial Intelligence in Health” [8]. However, ageism in AI extends beyond the confines of health care and health-related data and has been described as *digital ageism* [9]. Ageist attitudes, beliefs, and practices may be overt or covert; for example, these conditions may be created through the bias of omission or exclusion [10]. Although most commonly directed toward older people [11-14], ageism can also be directed at younger individuals [15]. The concept and extent of digital ageism, however, are not well established in the literature on bias in AI. This review aims to address this knowledge gap by examining bias in AI systems against older adults.

There is an increasing presence of technology and AI in our daily lives, with substantial applications in health care [16], education [11], employment [17-19], finance [20,21], and law [22,23], generating a “digital world” from the 2.5 quintillion bytes of data created daily [24]. However, due to structural barriers, such as limited internet access, older adults can be socially and digitally excluded [25,26]. The exclusion of older adults means that their needs and desires are not considered or reflected in the technology pipeline, spanning from hardware design [27-29] to AI systems development, which can negatively impact their desire to adopt the technologies [30,31]. For instance, in studies analyzing smartphone design and use, older adults are commonly excluded [32], and when they are included, they are classified into a broad and vague age category, such as “50+” or “60+” [33,34]. This can contribute to misconceptions held by developers that lead them to view older people as a monolith rather than a heterogenous group [35], particularly regarding ageist stereotypes in the technology design process that characterize aging as a state of inevitable decline that will require costly care [35-37]. Consequently, technology developers assume that older people will need and want health technologies to compensate for declining abilities [36], resulting in the development of technologies that are suboptimized for older adults’ abilities and needs. Cumulatively, a digital experience that is inaccessible and unrelatable is created [38].

Technologies that are created on the basis of inaccurate assumptions about older people can cause users (ie, older people) to internalize negative stereotypes, reducing their self-efficacy and willingness to engage with technologies in general [35]. A decreased use of technology by older adults compared to younger populations can disincentivize developers to consider older adults as end users for future designs [38], thereby contributing to a vicious cycle that excludes older adult and sustains ageism. The result of these multilayered barriers, including barriers to access and ageism throughout the technology development pipeline, is that older people collectively produce less data for AI training [39]. These imbalanced data sets with underrepresented key segments raise questions and concerns about how older people are perceived in the “digital world” and the implications of deploying ageist AI systems.

The goals of this study are interdisciplinary in nature and aim to explore how age-related bias is encoded and amplified in AI systems and understand any corresponding societal, legal, and ethical implications. This review will address the following research questions:

1. What is known about age-related bias in AI technology?
2. How do AI systems encode, produce, or reinforce age-related bias?
3. What literature exists on the extent of age-related bias in AI systems?
4. What is the state of knowledge on older people’s experiences of age-related bias in AI systems?
5. What are the social, legal, and ethical implications of age-related bias in AI systems?

This study will contribute to the global conversation about bias in AI systems and the associated concerns of fairness [40-44] by broadening the dialogue on race and gender biases to include the impacts of age-related bias on older people. The foundational knowledge gained through this study will be used to identify related challenges and opportunities in the subfield of AI and age-related bias, establish a multiphase research program aimed at defining ageism in AI, and develop a deeper understanding of ageism in the context of AI predictive modelling.

## Methods

### Methodology and Framework

This scoping protocol was developed using guidance from the PRISMA-ScR (Preferred Reporting Items for Systematic Reviews and Meta-Analyses Extension for Scoping Reviews) reporting guideline [45]. A scoping review methodology is optimal for our exploratory aims of synthesizing the evidence and assessing the scope of the literature on ageism in AI [45]. Bias assessment in AI is an emerging field, especially for age-related bias. The study will follow the methodological framework developed by Arksey and O’Malley [46] and further enhanced using the recommendations by Levac et al [47]. This framework has 6 stages that aim to achieve both in-depth and broad coverage of all the available literature [46]. This scoping review has been registered in the Open Science Framework (OSF) database [48].

### Step 1: Identifying the Research Question(s)

As scoping review questions are recommended to be broad [47], the research team approached the literature using an interdisciplinary lens to include legal, ethical, technical, and social perspectives and queries. The authors include gerontologists, legal scholars, engineers, ethicists, a computer scientist, a philosopher, and a public health graduate student. Collaborators on the project are philosophy scholars and members of provincial- and national-level Canadian organizations interested in aging and technology. Through discussion, the team generated the research questions stated above.

**Figure 1:**
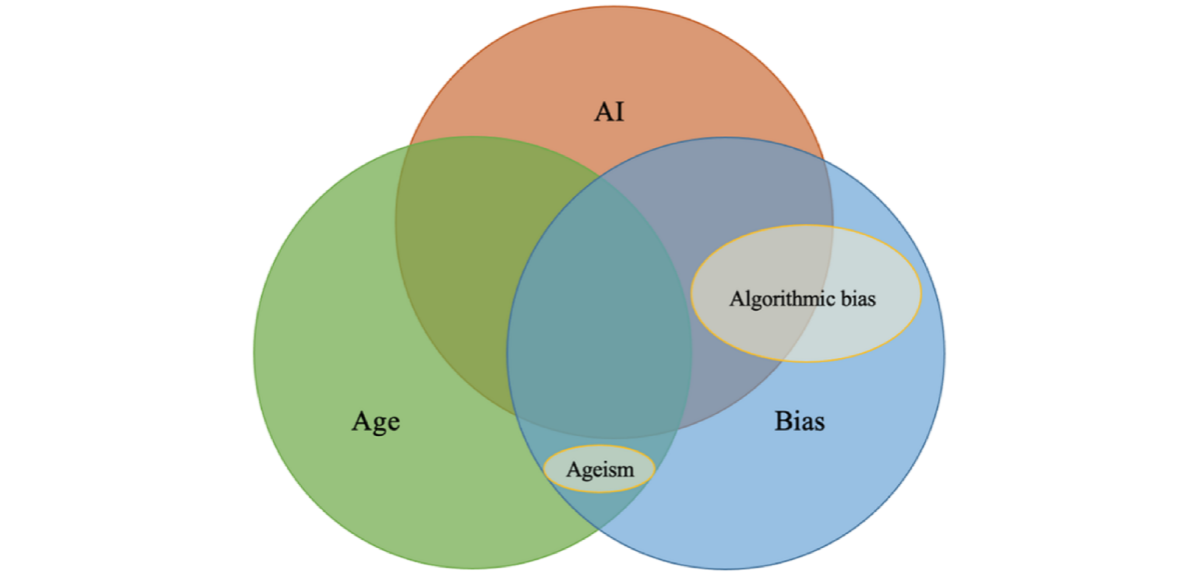
Main concepts included in the search strategy. AI: artificial intelligence.

Under an information specialist’s guidance, the research team developed a search strategy consistent with scoping review methodology [47]. The team and collaborators articulated three distinct concepts: (1) AI [1]; (2) age-related bias (ageism) [7]; and (3) algorithmic bias, defined as bias in the algorithms (Figure 1). In contrast to previous work [38], the search strategy for this study included all types of AI and its application across all devices used by humans (eg, AI used on mobile devices, computers) and encompassed multiple disciplines (eg, health-related, business) to ensure a comprehensive search.

### Step 2: Identifying Relevant Studies

#### Peer-Reviewed Literature

This section will describe the completed search strategy for the scoping review. The search strategy was informed by test searches in Scopus, Medline, IEEE Xplore, ACM Digital Library and Google Scholar with the key search terms “artificial intelligence” and “ageism.” The first 200 results in each database were screened by checking their title and abstract for relevant records. There were no relevant search results that explicitly discussed AI and ageism, so the concept “ageism” was expanded and changed to “age” to capture more records discussing aging as suggested by the information specialist. Next, individual key terms were searched to gather synonyms, and a synonym list was generated (see Multimedia Appendix 1). Due to the high number of “artificial intelligence” and “age” synonyms, these terms were categorized into broad topics under each term. For example, the 57 synonyms found for AI were categorized into synonyms that were specifically related to the following topics: AI techniques (eg, machine learning), general technology using or intersecting with AI (eg, big data, informatics, data science), and AI applications related to health technologies (eg, biomedical technology). The synonyms of age were categorized into terms related to bias (eg, age-related bias, ageist), older adults as a demographic or population (eg, aging person, seniors), and a field of study (eg, gerontology). The list of all the synonyms and their categories as well as their frequency of appearance in the searches can be found in Multimedia Appendix 1.

We conducted test searches by combining synonyms of our key concepts in Scopus, a multidisciplinary database that matched the nature of our study, to examine which synonym combinations could generate relevant records. After searching for all the synonyms proposed, we found 5 key papers that discussed age-related algorithmic bias, 53 relevant articles, and 29 additional synonyms occurring in the titles, abstracts, or keywords of these records (Multimedia Appendix 1). Based on the synonyms that provided the most relevant literature, the expanded search strategies were built based on the following synonyms: “machine learning,” “artificial intelligence,” “algorithms,” “neural networks,” “deep learning,” “algorithmic bias,” “biased,” “discrimination,” “ageism,” “age,” and “older people.” The themes of these synonyms were related to AI techniques, algorithmic bias, ageism, and age as a demographic. We revised our search strategies (Multimedia Appendix 1) and inclusion and exclusion criteria (Textbox 1) via the analyses of the key synonyms (Multimedia Appendix 1) identified in our test searches following further consultation with the research team, collaborators, and information specialist.

**Inclusion and exclusion criteria for the scoping review.**

**Inclusion criteria**

- Printed in English
- Peer-reviewed publications and conference papers
- Available electronically in full text
- Meet one of the two criteria below:
  Report “artificial intelligence” (algorithms that predict or classify data), “bias,” and terms related to “age” (aging, older, demographic)
  Report facial recognition and age or demographics

**Exclusion criteria**

- Theses and dissertations
- Conference abstracts and proceedings
- Perspectives and editorials
- Books and book chapters
- Letters to editors
- Manuscripts using nonhuman samples
- Manuscripts that do not use human data
- Children as the target population
- Theoretical analysis
- Mathematical formulations
- Nonhuman studies

The final search strategy was developed in Scopus and then translated to the other 5 databases (Web of Science, CINAHL, EMBASE, IEEE Xplore, and ACM digital library). As IEEE Xplore had limitations on the number of terms and wildcards used for the search, we iteratively tested one theme or combinations of themes using different subsets of the proposed synonyms. A synonym was deleted if its addition to the search did not produce relevant results. We screened the first 200 records produced in each testing and eliminated the corresponding synonyms if none of the results were relevant.

The search parameters included peer-reviewed publications and conference papers published in English and available electronically in full text. Due to the study’s interdisciplinary nature, we did not limit the study design for inclusion. The search strategy was also not restricted by publication date since the term “artificial intelligence” has existed for over 50 years [18]. The following sources were excluded to balance study breadth with feasibility and timeline limitations: theses, dissertations, conference abstracts, nonpeer-reviewed conference proceedings, perspectives, editorials, books, book chapters, and letters to editors. The results of the search strategy form a base for the next steps of our scoping review of ageism in AI.

#### Grey Literature

Given the anticipated paucity of academic research studies directly focused on ageism in AI, grey literature will increase the breadth and relevance of our findings. With the search strategy established, an iterative grey literature search strategy will be used to retrieve documents in the public domain that are relevant to any of our research questions to ensure that all relevant information about age-related bias in AI is captured. Grey literature will be retrieved by searching grey literature databases (OpenGrey and Grey Literature Report). Targeted searches of websites identified by the research team (eg, Algorithm Watch, Healthcare Information and Management Systems Society, The Centre for Data Ethics and Innovation) will also be conducted to retrieve documents such as white papers, policy papers, technical papers, and government reports. These documents will be downloaded in PDF form and added to a separate Microsoft Excel table to record the website source. After a thorough full-text review of each source, a rating scale of 0 to 4 (0=no reference to AI and ageism, 1=mentioned “age” in a list of types of biases, 2=one sentence related to the age-related bias, 3=two or three sentences related to age-related bias, 4=more than three sentences relevant to AI and ageism) representing the relevancy of the document was used to identify which sources were most relevant to the study. The included sources (anything with a rating above 0) had the relevant portions of text with the corresponding page numbers highlighted and documented, which will be themed by the research team according to each research question. To date, we have completed a preliminary manual Google search using the terms “artificial intelligence” and “ageism,” which identified 213 results in November 2021. A reviewer (JS) from the research team opened each web page to screen the content on the page for relevance. We found additional pages from law-related blogs that referenced employment discrimination related to age-related algorithmic bias.

Given the anticipated legal and ethical implications of ageism in AI, a review of relevant legislation, regulations, and jurisprudence (court cases) will be used to augment our academic and grey literature searches. These data sources will address research question 5 (What are the social, legal, and ethical implications of age-related bias in AI systems?). This process will be led by the team’s legal scholars and focused on understanding the legal and regulatory framework to protect and prevent age-related bias and unjust discrimination in AI. The iterative legal search strategy will begin with a review of relevant secondary sources including legal dictionaries and encyclopedias, followed by a review of legal treatises, law reviews and journals, statutes, and administrative regulations, and finally an analysis of the relevant case law. The legal databases WestlawNext Canada and CanLII will be canvassed in this legal review. Given the relative novelty of AI in the legal realm, a broad keyword search will be used to capture the relevant material. The keywords include “artificial intelligence;” “A.I.”; “machine learning”; “ageism”; and “discrimination.” The keyword search will be periodically refined to limit search results to various legal domains, including employment law, human rights law, and health law.

### Step 3: Study Selection

The search results will be exported into Covidence, a commonly used web-based literature review tool. The eligibility of the publications was determined based on a screening guideline established by 2 reviewers (JS and CHC; Textbox 1) and pilot-tested on 20 titles and abstracts. An article meets the inclusion criteria if its abstract reports “artificial intelligence” (eg, predict or classify data), “bias,” and terms related to “age as a population” (eg, aging, older, demographic). Any articles about facial recognition will be included if they mention age or demographics. We consider the risks of bias as being high in facial recognition, even without the explicit reporting of “bias,” because research has demonstrated an algorithmic bias of facial analysis technology among older adults with dementia [49]. Once duplicates are removed, the titles and abstracts of all remaining articles will be screened by 2 independent reviewers using the screening guideline developed. The reviewers will meet at the start of the screening process to finalize and clarify the inclusion criteria and convene shortly after the screening commences to refine the criteria. The full text of each included citation will be reviewed by 2 independent reviewers to determine the article’s relevance to the primary research questions of this study. If disagreements among reviewers cannot be resolved through discussion, the principal investigator (CHC) will make the final decisions for study selection. We will hold regular biweekly meetings to discuss the results.

### Step 4: Charting the Data

We will chart the data based on the primary research questions using tools such as Google sheets or Covidence. Textbox 2 represents a sample format for data charting. To test the extraction forms for both academic and grey literature, the reviewers will independently chart the data of 5 to 10 included sources. Once interrater reliability is established, the extraction forms will be distributed to all the team members. For 20% of the included academic and grey literature sources, a second reviewer will verify the extraction. As data charting is an iterative process, we expect the team may modify elements of the forms so that they reflect the relevant findings of the articles included.

**Sample data that will be charted.**

**Article information**

- Article title
- Data charted by (initials)
- Author(s)
- Year
- Country
- Aim or purpose
- Study design

**Artificial intelligence (AI)**

- Branch of AI
- Algorithms as described
- Type and source of data

**Population**

- Does the article report age as demographic information of the study population?
- Does the article report on the experience of older people with age-related bias?

**Bias identification and attribution**

- Data set: yes or no
- AI algorithm: yes or no
- Methods proposed to mitigate bias, if any

**Implications**

- Legal implications
- Societal implications
- Ethical implications

### Step 5: Collating, Summarizing, and Reporting the Results

Data charting will serve as the first step to summarizing the results. We will record each study based on fundamental information including article title, author(s), publication year, country, and study aims. Based on what is commonly reported in other AI reviews, we will potentially include technology-related information such as the aim of the technology, stage of the technology development, data used, and validation methods. To synthesize the findings, we will conduct a thematic analysis and use a narrative description to describe the work according to study design (quantitative or qualitative), any emerging patterns identified, ethical implications, as well as legal considerations. Collation of the findings will inform gaps for future studies in the field of AI and ageism.

### Step 6: Consultation

To allow for stakeholder involvement and additional insights beyond the literature, the preliminary summary document will be circulated to stakeholders, including our national and international research collaborators with expertise or interests in aging, subject experts from the Temerty Centre for Artificial Intelligence Research and Education in Medicine at the University of Toronto, a senior’s advocate, and older adults. These stakeholders have been involved from the early stages of the research conceptualization as knowledge users on our grant application.

## Results

The database searches resulted in 7595 records when the searches were piloted in November 2021. Data will be abstracted in a tabular format to support drafting of a narrative summary. A scoping review publication will serve as the main presentation of the findings. The remaining stages of the search is proposed to reach completion by December 2022.

## Discussion

The findings of this review will provide foundational information to advance our understanding of the concept and extent of digital ageism, which occurs when technologies deliberately or inadvertently exclude older adults, prioritize younger adults, or fail to recognize the diverse needs of the older adult demographic through various means [9]. The results from this study will provide interdisciplinary insights about digital ageism and the ways in which it is perpetuated in AI systems, such as from a lack of representative data sets (ie, data disparity). Overlooking older people prevents them from enjoying the full benefits of AI-based technologies and innovations, which can reinforce societal biases and inequity in our increasingly digital society.

A strength of our review is that our study is interdisciplinary and will shed light on AI and age-related bias regarding older adults from societal, legal, ethical, and technical perspectives. We have a rigorous methodology based on a scoping review framework and a comprehensive search strategy that includes interdisciplinary and discipline-specific databases. A team of researchers from different fields will interpret and generate findings that will foster further discussions and provide a direction for future work related to AI and older adults. One of the potential limitations of this study is the exclusion of publications in non-English languages as well as studies that do not discuss bias or age-related bias explicitly, potentially excluding research that unknowingly uses skewed data due to age-related bias embedded in specific AI algorithms. The inclusion of literature that explicitly discusses or recognizes the potential for age-related bias allows us to answer our current research questions. Our future work will explore the presence of implicit age-related bias in AI, as well as how ageism is reflected in a subset of AI algorithms.

To our best knowledge, this is the first scoping review to explore how age-related bias is encoded or amplified in AI systems and consider the societal, legal, and ethical implications. This scoping review protocol documents the search strategy and outlines the in-depth process for our rigorous synthesis of the literature on AI and ageism. Once the review is complete, we will connect with organizations at provincial, national, and international levels to discuss the findings and build the corresponding interview guides for in-depth semistructured interviews. Our review has the potential to establish the intersection of AI and ageism, advance knowledge about digital ageism, and inform future regulation and policy in this currently uncharted territory.

## Appendix

Multimedia Appendix 1 Search strategies and synonyms.

## Abbreviations

AI: artificial intelligence
OSF: Open Science Framework
PRISMA-ScR: Preferred Reporting Items for Systematic Reviews and Meta-Analyses Extension for Scoping Reviews
