# Supplementary material for: Ageism and Artificial Intelligence: Protocol for a Scoping Review
Source: JMIR Res Protoc. 2022 Jun 9;11(6):e33211. doi: 10.2196/33211 (PMC9227654; doi:10.2196/33211)
Supplement: Multimedia Appendix 1 [file resprot_v11i6e33211_app1.docx]

**Ageism and Artificial intelligence: A Protocol for a Scoping Review**

**Authors**

Charlene H. Chu RN, GNC(c), PhD ^1,2,3^

Kathleen Leslie RN, JD, PhD ^4,5^

Jiamin Shi, BSc, MPH ^1,6^

Rune Nyrup, PhD ^7^

Andria Bianchi, PhD ^3,6,8^

Shehroz S. Khan, PhD ^3,9^

Samira Rahimi Abbasgholizadeh, BEng, PhD ^10,11^

Alexandra Lyn BEng, JD, MBA, LLM ^4^

Amanda Grenier, BSW, MSW, PhD ^2,12,13^

^1^ Lawrence S. Bloomberg Faculty of Nursing, University of Toronto, Toronto, ON
^2^ Institute for Life Course and Aging, University of Toronto, Toronto, ON
^3^ KITE-Toronto Rehabilitation Institute, University Health Network Toronto, ON  ^4^ Faculty of Health Disciplines, Athabasca University, Athabasca, AB

^5^ Canadian Health Workforce Network, Ottawa, ON

^6^ Dalla Lana School of Public Health, University of Toronto, Toronto, ON

^7^ Leverhulme Centre for the Future of Intelligence, University of Cambridge, Cambridge, UK

^8^ Department of Bioethics, University Health Network, Toronto, ON

^9^ Institute of Biomedical Engineering, University of Toronto, ON

^10^ Department of Family Medicine, McGill University, Montreal, Canada

^11^ Mila - Quebec AI Institute, Montreal, Quebec, Canada

^12^ Factor-Inwentash Faculty of Social Work, University of Toronto, ON

^13^ Baycrest Hospital, Toronto, ON

**Corresponding Author:** Charlene H. Chu, RN, PhD

University of Toronto - Lawrence S. Bloomberg Faculty of Nursing,

155 College St Suite 130, Toronto, ON M5T 1P8

416-946-0217

[charlene.chu@utoronto.ca](about:blank)

# **Supplementary File** – Screenshots of search strategies

Scopus


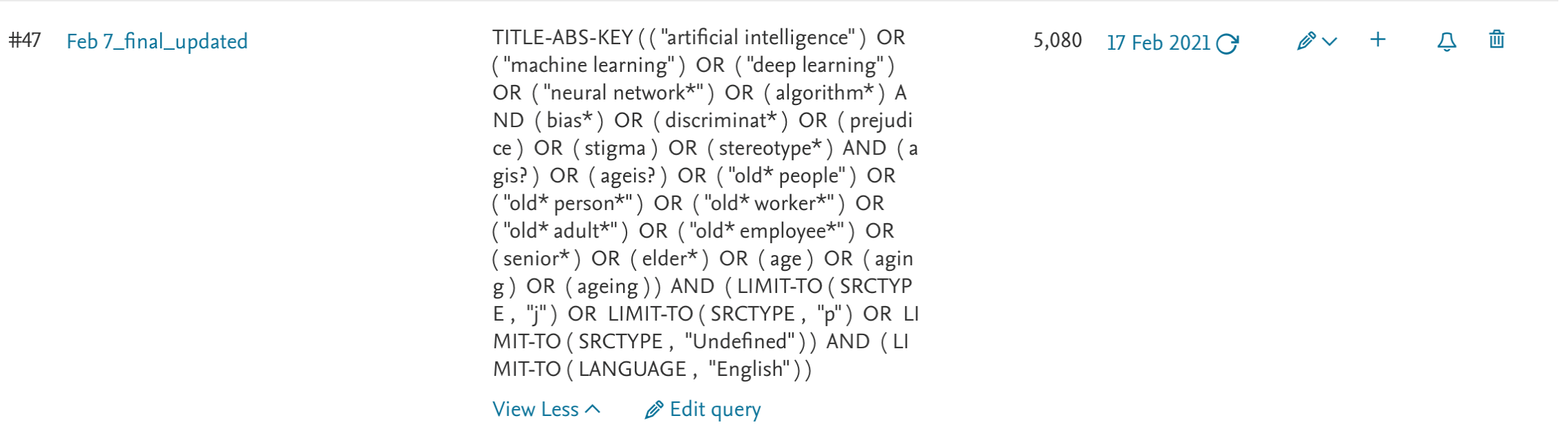


Web of Science

#1 AND #2 AND #3


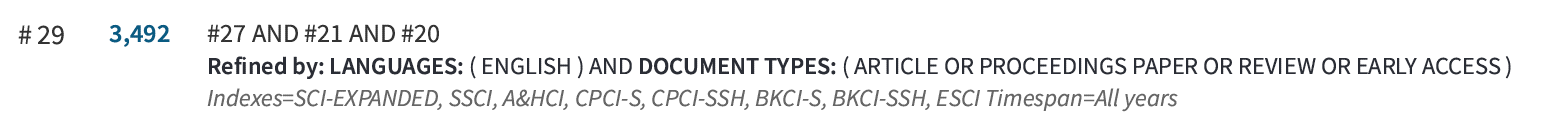


#3


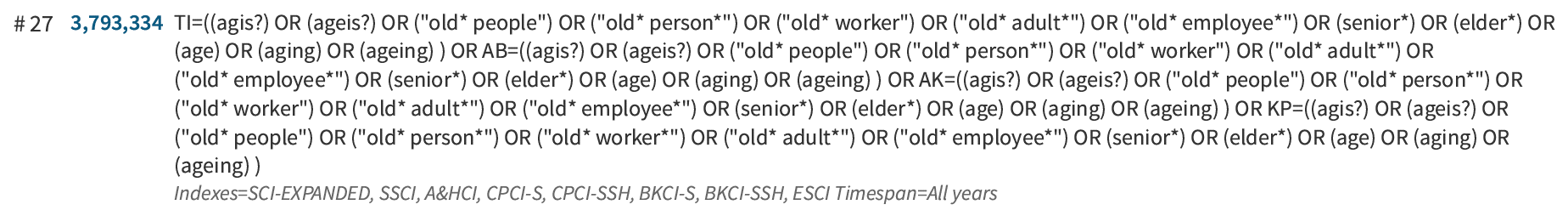


#2


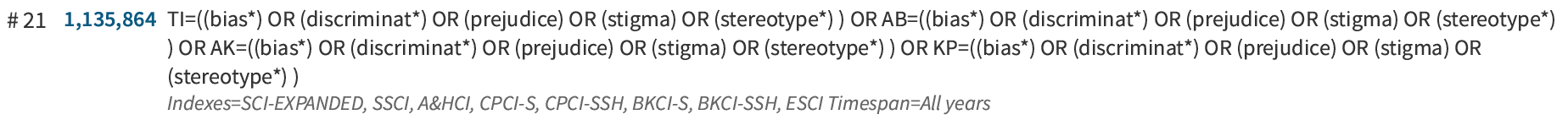


#1


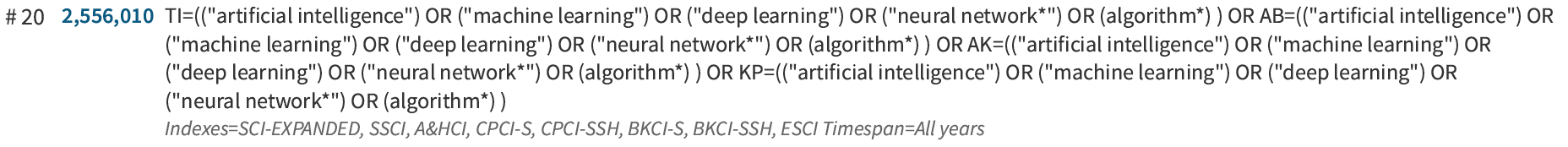


CINAHL


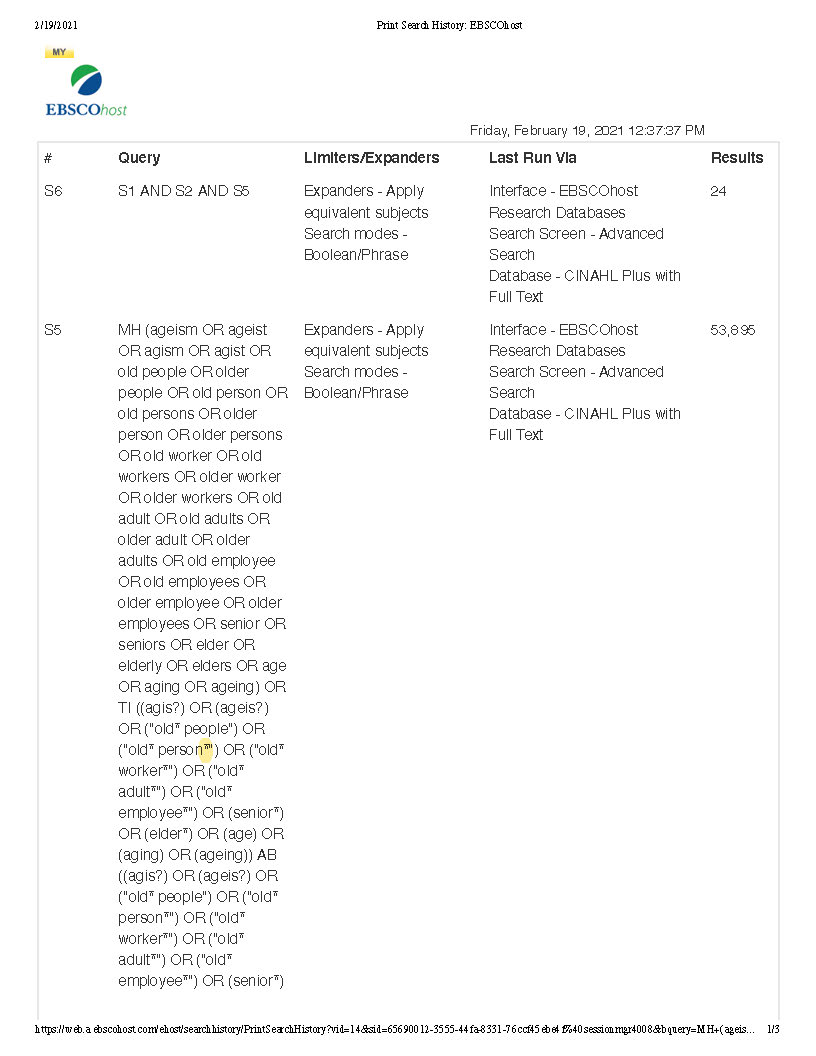


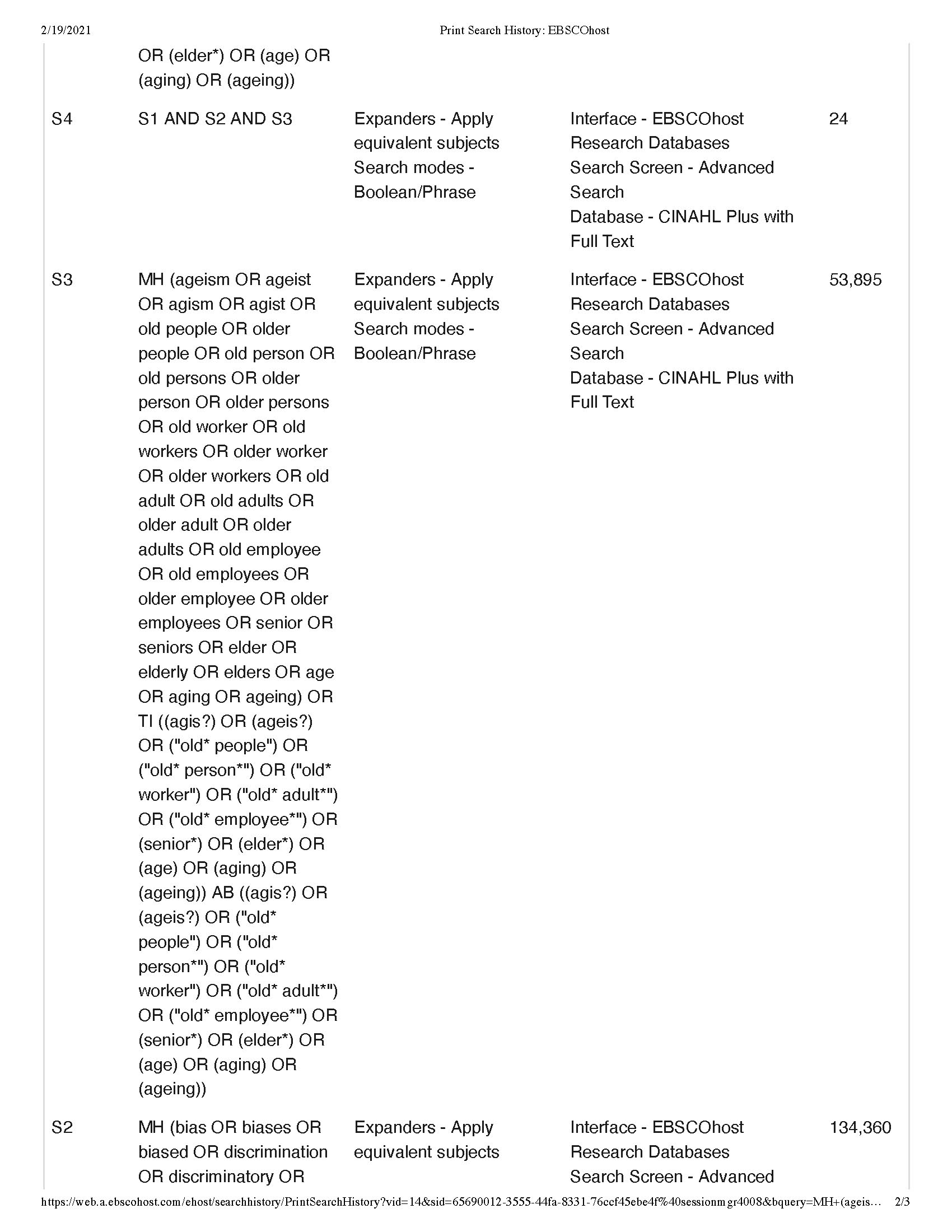


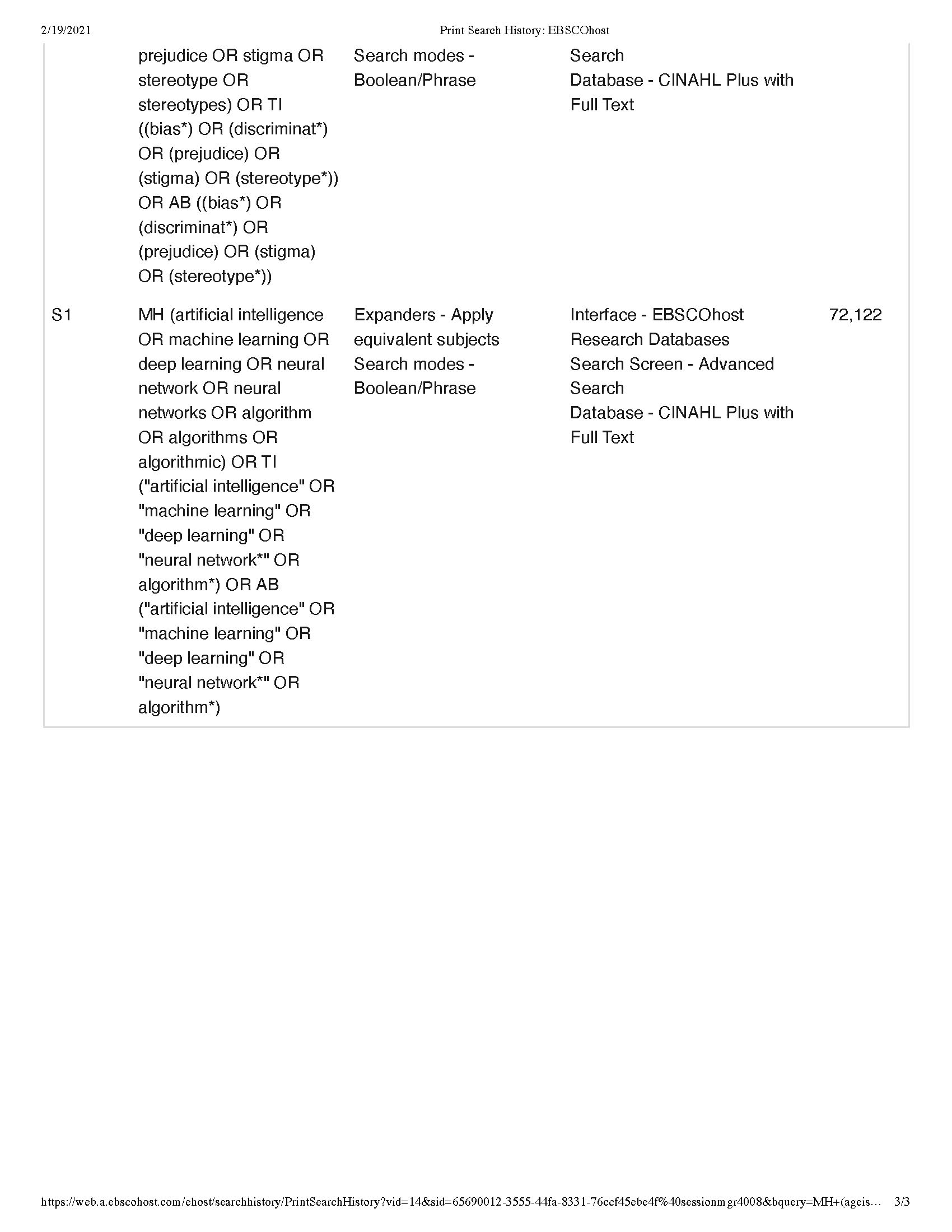


EMBASE

**
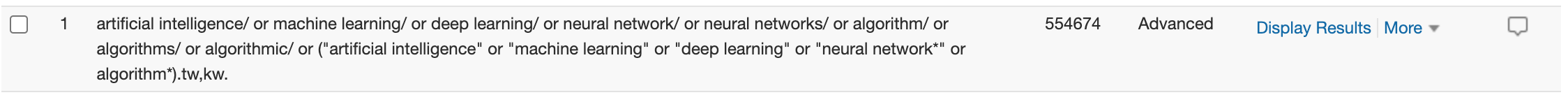
**


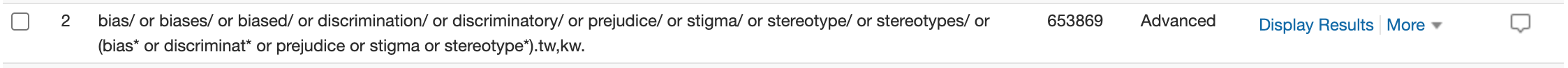

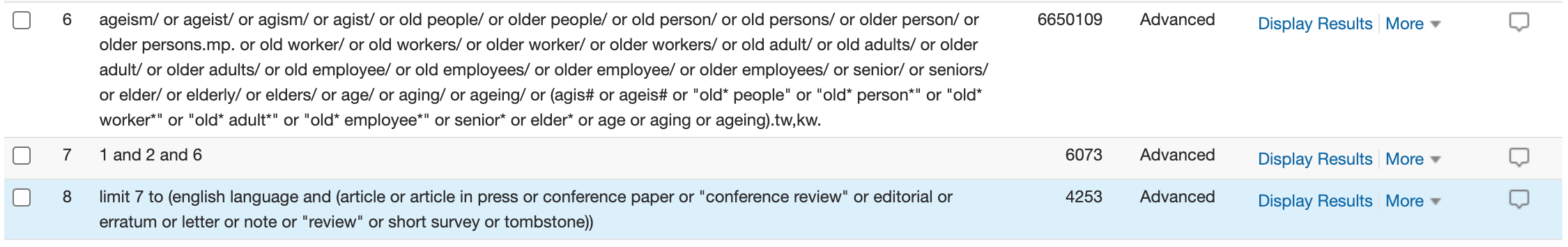


IEEE

**
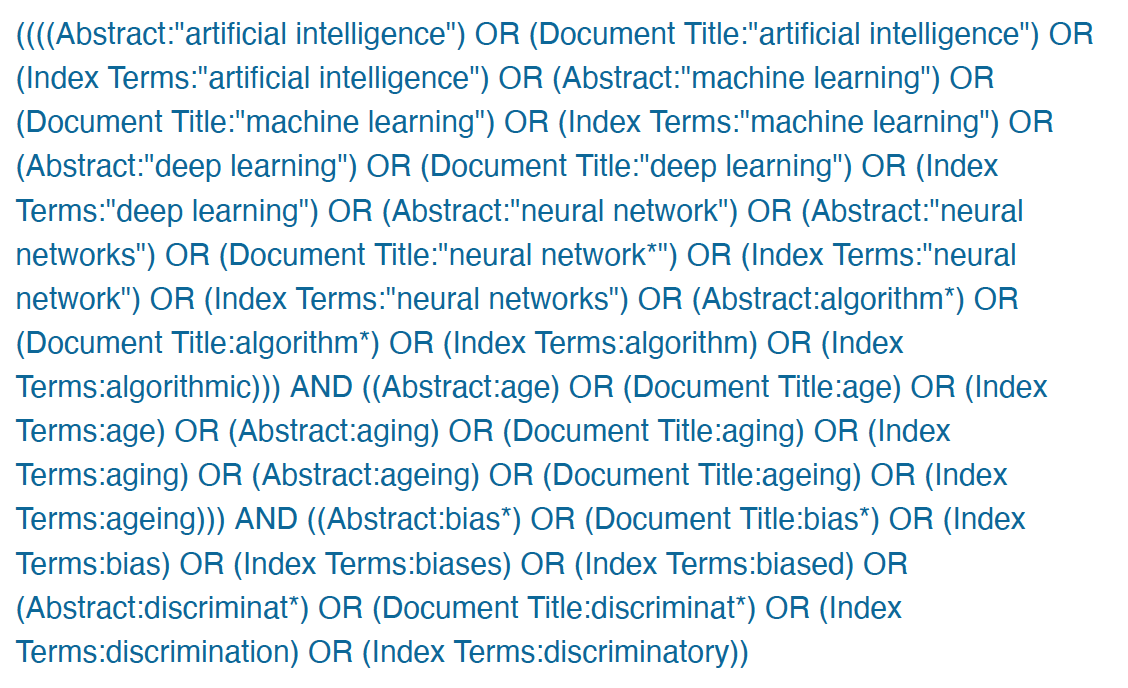
**

ACM digital library

**
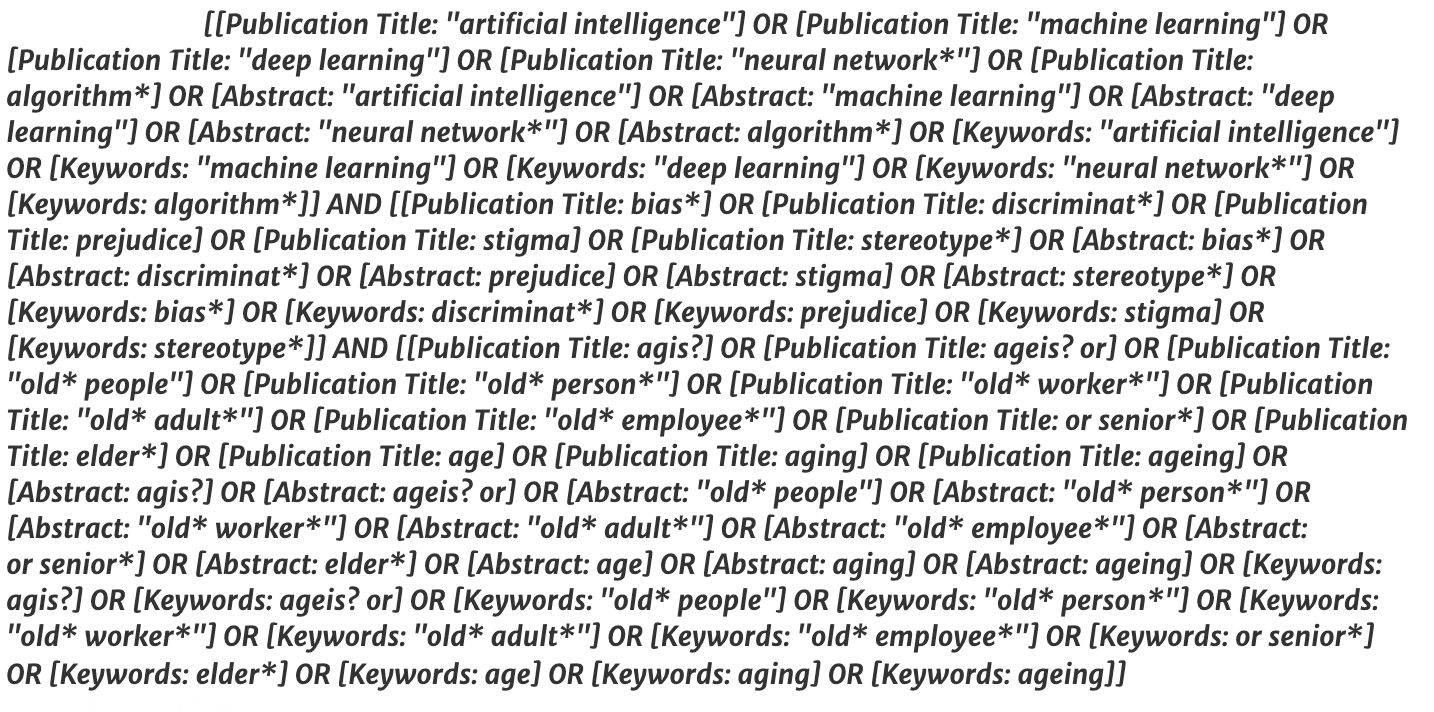
**

Synonyms of artificial intelligence (AI)

| **AI techniques** | **Broad, technology related terms** | **Health technology** | **AI and Bias** |
| --- | --- | --- | --- |
| - artificial intelligence - machine learning - deep learning - support vector machine - multifactor dimensionality reduction - neural networks - AI and intelligence - semisupervised learning - semi-supervised learning - intelligent machine - latent class model - machine intelligence - imitation of human intelligence - intelligent computer - simulation of human intelligence - development of 'thinking' computer systems - computational intelligence | - applications - big data - computers - digital future - data aggregation - meta heuristics - data mining - data science - heuristics - natural language processing - pattern recognition, automated - radiographic image interpretation, computer-assisted - signal processing, computer-assisted - image recognition - intelligent technology - diagnosis, computer-assisted - algorithms - chatbox - sentiment analysis - speech recognition - word embeddings - word vector | - biomedical technology - medical informatics - medical informatics - digital health - mobile health - telemedicine - drug therapy, computer assisted - therapy, computer-assisted - genetic algorithm | - algorithmic bias - algorithm bias - algorithmic discrimination - algorithm discrimination - ageist algorithm - AI-driven discrimination - automation bias - model bias - flawed algorithm - biased algorithm - demographic bias |

# Synonyms of Age

| **Ageism/Age-related bias** | **Age referring to a demographic or a population** | **Age as a field** |
| --- | --- | --- |
| - bias in age - ageist stereotypes - ageist - age discrimination - age-based bias - age bias - age prejudice - agism - age-related discrimination - age-based discrimination - discrimination on the basis of age - age stereotype - stereotype of ageing - age-related stigma - stigma of age - auto-ageism | - older worker - ag(e)ing - older persons - older adults - seniors - elderly - elders - senior citizens - older employee - ageing workforce | - Gerontology - geriatrics - ageing studies |

# Synonym analysis

1. Frequency analysis of synonyms

- The search generated 53 relevant articles. From these, we identified 29 additional synonyms and from the titles, abstracts, or key words of these papers.
- We compared the searched synonyms for each of these papers
- A table detailing the frequency of each synonym was create
- Based on the following table, the top 10 synonyms are: machine learning, artificial intelligence, neural networks, deep learning, algorithms, age, older people, discrimination, biased, algorithmic bias.
  - AI-related: machine learning, artificial intelligence, algorithms (broad term), neural networks, deep learning
  - Algorithm & bias: algorithmic bias
  - Bias-related: discrimination, biased
  - Age-related: older people, age

Table 1. Frequency of synomyns and their occurance

**
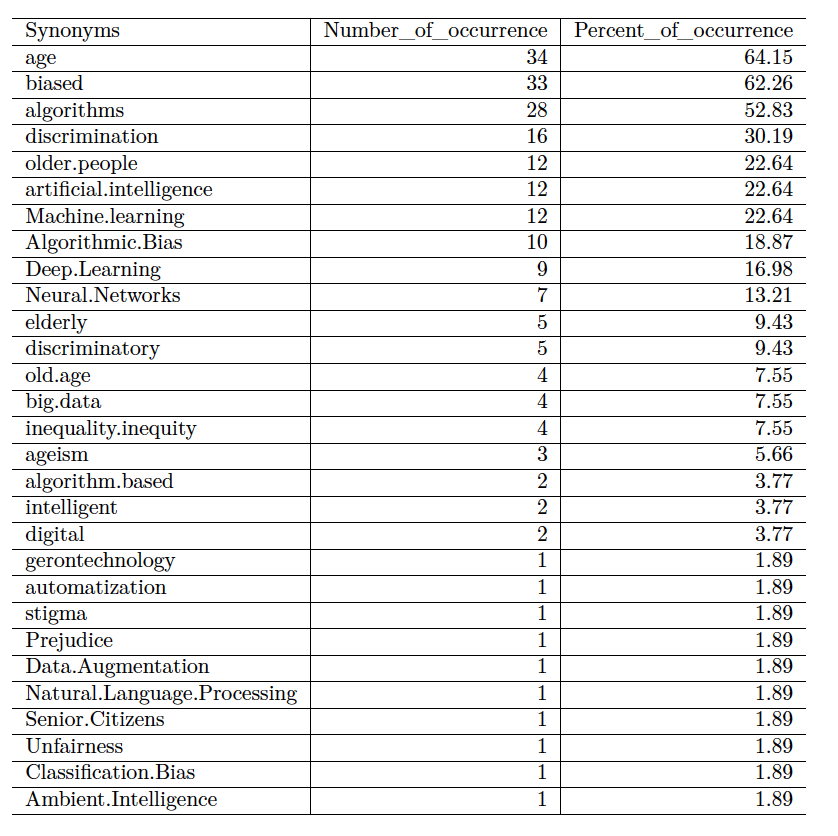
**

Figure 1. Visualization based on frequencies

**
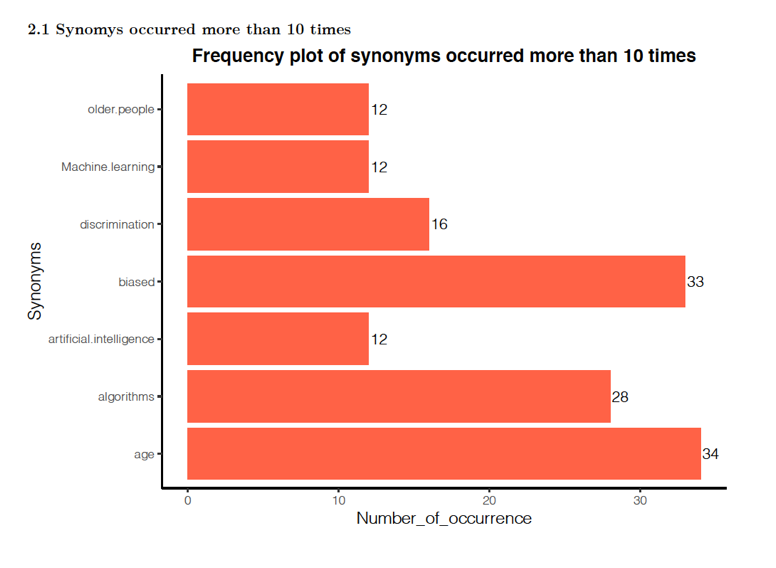
**

**
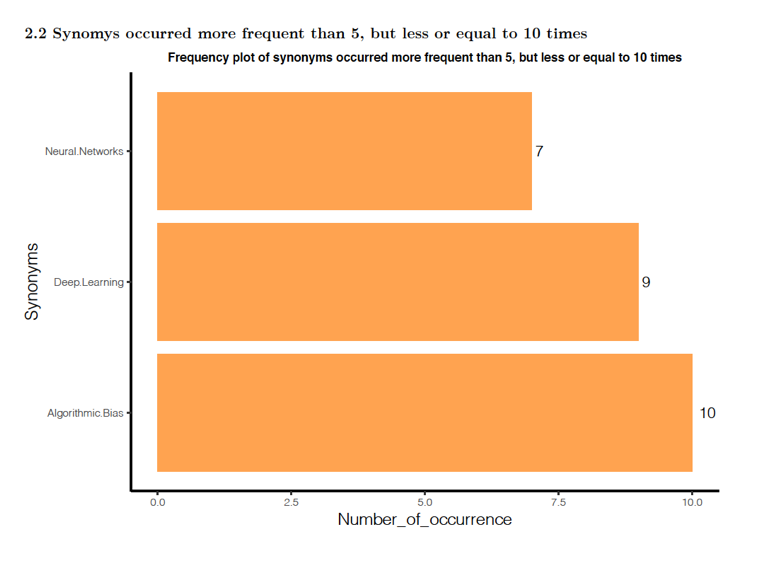
**

**
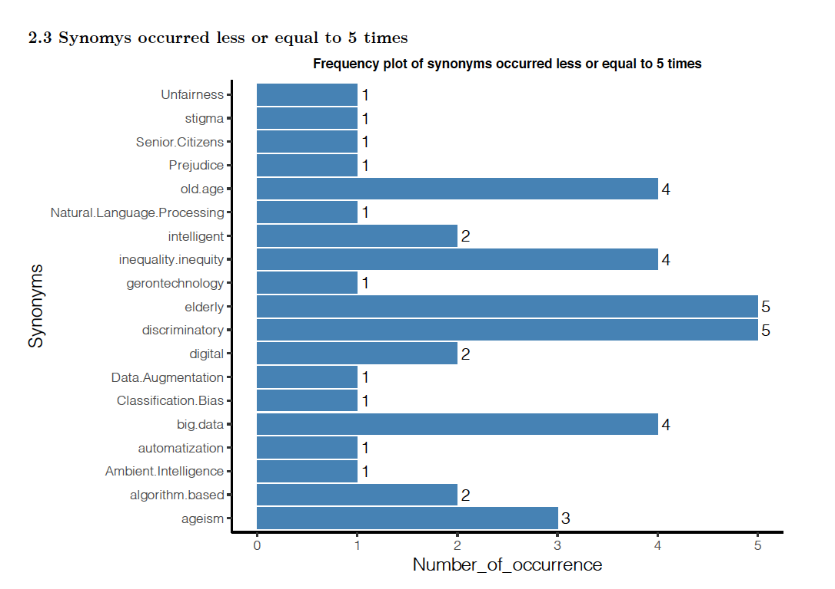
**
